# Consumer acceptance of reduced sodium white and multigrain bread: Impact of flavor enhancement and ingredient information on sample liking

**DOI:** 10.1111/1750-3841.16395

**Published:** 2022-12-05

**Authors:** Aubrey N. Dunteman, Soo‐Yeun Lee

**Affiliations:** ^1^ Department of Food Science and Human Nutrition University of Illinois at Urbana‐Champaign Champaign Illinois USA

**Keywords:** consumer, food additives, sensory analysis

## Abstract

Chronic consumption of sodium in quantities exceeding recommendations has led to sodium being designated as a nutrient of health concern for overconsumption. As a result of the prevalence of sodium overconsumption, the Food and Drug Administration (FDA) released voluntary sodium reduction goals for a wide variety of products on both short‐ and long‐term timespans. As food palatability may decrease when sodium is reduced, flavor enhancers such as monosodium glutamate (MSG) may provide a promising solution to mitigate such palatability loss. The objective of this research was to investigate consumer acceptance of white and multigrain breads with either a 43% or 60% reduction in sodium content and with and without MSG as well as to investigate the influence of information on consumer acceptance of these breads under blind, informed, and informed with education conditions. Seventy‐eight frequent bread consumers participated in the evaluations. A significant difference was evidenced across breads with different levels of sodium content and MSG status, although no difference was seen across the different evaluation conditions. Consumer segmentation found multiple consumer clusters showing different liking patterns of the bread treatments for both white and multigrain breads. Breads with sodium content set at the FDA's long‐term goal with and without MSG were liked no differently in nearly all attributes evaluated than the full‐sodium bread demonstrating the feasibility of producing acceptable reduced‐sodium breads. Future research characterizing the predominant sensory attributes of full‐sodium and reduced‐sodium breads with and without MSG would be valuable for identifying the drivers of liking in such products.

**Practical Application**: The findings of our study suggest that consumer liking of reduced sodium white and multigrain breads could be improved with the addition of monosodium glutamate. Increasing the acceptance of reduced sodium food products could help to reduce the risk of hypertension and subsequently heart attacks and stroke for the American population.

## 1 INTRODUCTION

Ninety‐seven percent of Americans over the age of one consume sodium in quantities greater than their age and gender's recommended intake level (Moshfegh et al., 2005). The average sodium intake for American adults currently averages approximately 3400 mg daily, exceeding both the adequate intake (AI) of 1500 mg and the chronic disease risk reduction intake (CDRR) of 2300 mg (National Academies of Sciences, Engineering, and Medicine, 2019). As a result of chronic consumption of sodium in quantities exceeding recommendations, it has been designated as a nutrient of health concern for overconsumption (Dietary Guidelines Advisory Committee, 2015). Research has shown that there is an association between higher sodium intake and a higher risk of developing hypertension which, if acquired, further increases one's risk for conditions such as coronary heart disease, congestive heart failure, stroke, and renal disease (Hermansen, 2000; Kim & Andrade, 2016; Maluly et al., 2017). With the chronic overconsumption of sodium a likely contributor, 56% of American adults suffer from either hypertension or prehypertension (FDA, 2022) and the estimated lifetime risk of developing hypertension is hovering at 90% (Dietary Guidelines Advisory Committee, 2015).

One of the main staple foods seen in diets across the world is bread. Due to the prevalence of daily bread consumption and the moderately high sodium content that accompanies it, bread has become a major contributor to dietary sodium intake (Bolhuis et al., 2011). Average sodium contribution from bread ranges largely across the world; in Ireland, sodium contribution from bread ranges from 15% to 26%, whereas in Kuwait, bread contribution is as high as 34% (Food Safety Authority of Ireland, 2005; Irish Universities Nutrition Alliance, 2016; Jawaldeh & Al‐Khamaiseh, 2018). As a result of the high rates of sodium overconsumption, the Food and Drug Administration (FDA) has developed sodium reduction guidelines for a variety of food products, including bread. In the short term of 2 years, the FDA recommends sodium content in white bread varieties to be reduced 16%, while in the long‐term of 10 years, they recommend the sodium content to be reduced 43% (Food and Drug Administration, 2016). In 2021, the FDA released an updated document detailing voluntary sodium reduction guidelines focusing solely on short‐term 2.5‐year goals (Food and Drug Administration, 2021). This reduction in sodium is achieved by reducing added salt from the formulation, which will subsequently impact technological processes involved in bread production and final product characteristics. Despite the many functions that salt holds in bread production, such as textural improvement and yeast control (Markus et al., 2012), minimal research has been conducted on determining the level at which salt can be reduced before a decrease in quality is perceived and if flavor enhancers can mitigate such drawbacks associated with less salt.

Consumer perception of reduced‐sodium bread may already be at a disadvantage due to prior assumptions about foods with lower sodium content. Research on nutritional labeling of bread found that roughly 38% of consumers expect reduced taste in rolls labeled as having low salt content and that salt content was relatively important to 27% of consumers (Gębski et al., 2019). Conversely, researchers have found that consumer willingness to pay for bread with low sodium content has been valued at 20% over the price of normal bread, indicating the absence of salt is seen as a superior feature for bread by some (Di Vita et al., 2016). Without advertisement of bread containing reduced‐sodium content, consumers sensory perception may not be affected with a 10% reduction in salt or greater at nearly 20% when accomplished through two cumulative reduction steps (Antúnez et al., 2016).

Main obstacles cited by those trying to reduce their sodium consumption include complaints related to a reduced palatability of the food made with less salt (de Freitas Agondi et al., 2012). The salts of glutamic acid, including calcium di‐glutamate and monosodium glutamate (MSG), have been accepted internationally as flavor enhancer food additives (Carter et al., 2011; Jinap & Hajeb, 2010). MSG is a particularly promising flavor enhancer for its ability to enhance the palatability of reduced sodium foods as salty and savory tastes are intensified when present (Maluly et al., 2017; Okiyama & Beauchamp, 1998; Roininen et al., 1996). Many unsubstantiated health concerns have been commonly regarded as symptoms of MSG consumption leading to misconception in much of the general population. Claims of various side effects have been shown unrelated to MSG consumption as no scientific data has linked the symptoms to the ingredient (Walker and Lupien, 2000). Despite the concerns that have been raised about potential health issues and MSG, its safety has been demonstrated by multiple regulatory agencies, including the FDA and the Joint Food and Agriculture Organization of the United Nations/World Health Organization Expert Committee on Food Additives (JECFA) and it has been allocated Generally Recognized as Safe (GRAS) status (Maluly et al., 2017).

Lack of consumer understanding in relating to food additives such as MSG can have a negative impact on the perception and acceptance of food products. Respondents in a variety of studies have found that many consumers are highly concerned about food additives, with certain populations perceiving them as a significant hazard to food safety (Kang et al., 2021; Moravejolahkami et al., 2020; Shim et al., 2011). Training and consumer education have led to marked changes in attitudes toward food additives and have been demonstrated to improve consumer perceptions on the safety and applications of additives in foods (Lee et al., 2014).

Little research has been done on incorporating MSG in traditional sliced bread for sodium reduction purposes and its resulting impact on consumer perception; this study intends to address this literature gap while also investigating the impact of consumer education on such breads. The primary objective of this study was to investigate consumer acceptance of white and multigrain breads with varied levels of sodium and flavor enhancers. The secondary aim of this study was to investigate the influence of information on consumer acceptance of white bread with varied levels of sodium and flavor enhancers under blind, informed, and informed with selected education conditions. It was hypothesized that breads containing MSG would increase the consumer acceptability of reduced sodium breads when evaluated under blind conditions. It was also hypothesized that breads containing MSG would be less liked under informed conditions compared to breads without MSG. Finally, it was hypothesized that after undergoing the consumer education, consumer acceptance of breads containing MSG would increase.

## 2 MATERIALS AND METHODS

### 2.1 Consumer test panelists

The panelists for the consumer test were recruited primarily through e‐mail and a university newsletter. Many panelists were students, faculty, staff, and other members of the Champaign‐Urbana, Illinois area. Requirements for participation consisted of being at least 18 years of age, being a frequent consumer of bread (more than once per week), having no known food allergies or restrictions, and not being pregnant or nursing. In total, 78 panelists were recruited in this study (76% female, 24% male; 66% [18 to 25 years], 23% [26 to 35 years], 5% [36 to 45 years], 5% [46 to 55 years], 1% [56 to 65 years]). Females in this study were younger than males on average, with 78% of females between the ages of 18 and 25, and 53% of males between the ages of 26 and 35. This experimental protocol was reviewed and approved by the Institutional Review Board.

### 2.2 Experimental samples

White bread and multigrain bread loaves were formulated to contain various levels of sodium chloride (NaCl) and MSG. For white bread, samples prepared included one treatment containing the average amount of sodium for bread, two breads with reduced sodium content adjusted for the FDA's 2016 long‐term goal (43% reduction) where one is with and one is without added MSG, and two breads with reduced sodium content adjusted greater than the FDA's 2016 long‐term goal (60% reduction) where one is with, and one is without added MSG. For multigrain bread, samples prepared include the treatments detailed above with an additional bread at each level of sodium reduction containing a higher level of MSG. Table 1 shows the respective sodium content, NaCl content, and MSG content included in the formulation by flour weight. Bread samples were prepared in a Cuisinart CBK‐200 Convection Bread Maker (Cuisinart©, East Windsor, NJ, USA) using the Rapid Bake function.

**TABLE 1:** Sodium and monosodium glutamate (MSG) content in bread samples by flour weight

	Sample abbreviation	Sodium reduction (%)	Sodium (mg)	Sodium from NaCl and MSG^a^ (mg)	Sodium from NaCl (mg)	NaCl^c^	Sodium from MSG	MSG^c^ (mg)
Full sodium^b^	CTRL	0	615	590	590	1501	0	0
Reduced sodium long‐term	LT	43	350	325	325	826	0	0
Reduced sodium long‐term + MSG	LTMSG	43	350	325	311	791	14	112
Reduced sodium long‐term + MSGx2	LTMSG2	43	350	325	306	779	19	152
Reduced sodium extra‐term^d^	XT	60	246	221	221	562	0	0
Reduced sodium extra‐term + MSG	XTMSG	60	246	221	202	514	19	152
Reduced sodium extra‐term + MSGx2	XTMSG2	60	246	221	197	501	24	192

^a^ Ingredient abbreviations are as follows: NaCl, sodium chloride; MSG, monosodium glutamate.

^b^ Full sodium is based on the value of 1.5 g (590 mg sodium) of salt and 4.6 g (25 mg sodium) nonfat dry milk per 100 g of flour.

^c^ NaCl and MSG content are determined by using the total sodium content to calculate sodium with a 12.5% contribution from MSG and a 39.3% contribution from NaCl and selecting the amounts that will create a maximum of 0.76 g of MSG per 2 lb. loaf as preliminary baked breads indicate values over 0.75 g were perceived as too savory.

^d^ “Extra”‐term reduction goal was determined by combining the FDA's long‐ and short‐term goals for white bread and rounding up to 60%.

### 2.3 Sample production

The white bread loaves were composed of bread flour (Gordon Food Service®, Grand Rapids, MI, USA), nonfat powdered dry milk (Wal‐Mart Store, Inc., Bentonville, AR, USA), unsalted butter (Walmart Inc., Bentonville, AR, USA), MSG fine crystals (Ajinomoto North America, Inc., Itasca, IL, USA), granulated sugar (Domino Foods, Inc., Yonkers, NY, USA), vital wheat gluten (Bob's Red Mill Natural Foods, Inc., Milwaukie, OR, USA), rapid rise yeast (ACH Food Companies, Inc., Memphis, TN, USA), vitamin C (Pure Organic Ingredients, American Fork, UT, USA), and 95 ^o^F water. The multigrain bread loaves were composed of all the ingredients used in producing the white bread loaves, with the addition of whole wheat flour (The King Arthur Flour Company, Inc., Norwich, VT, USA) and 5 grain cereal (Azure Standard, Dufur, OR, USA). Ingredients were loaded into the bread pan by order of liquid ingredients, followed by dry ingredients, and lastly yeast.

Tables 2 and 3 describe the formulation for the white and multigrain bread loaves, respectively. A full sodium loaf was tested with a sodium content of 1.5 g per 100 g flour. Bread loaves were prepared less than 24 h prior to evaluation. Once the loaves cooled completely, they were stored in gallon‐sized Ziploc bags and cut into 25‐mm‐thick slices the day of evaluation. Bread samples were labeled with 3‐digit numerical codes to avoid potential panel bias.

**TABLE 2:** Ingredient compositions of white bread products

	Ingredient (%) w/w									
Sample	Bread flour	Water	Unsalted butter	Nonfat dry milk	Granulated sugar	Rapid rise yeast	Vital wheat gluten	Vitamin C	Salt	Monosodium glutamate
CTRL	54.44	33.75	4.35	2.5	2.29	1.01	0.73	0.1	0.82	0
LT	54.64	33.87	4.37	2.51	2.29	1.02	0.74	0.1	0.45	0
XT	54.72	33.92	4.38	2.52	2.3	1.02	0.74	0.1	0.31	0
LTMSG	54.61	33.86	4.37	2.51	2.29	1.02	0.74	0.1	0.43	0.060
XTMSG	54.68	33.9	4.37	2.52	2.3	1.02	0.74	0.1	0.28	0.083

Sample abbreviations are as follows: CTRL, full sodium; LT, reduced sodium long‐term; XT, reduced sodium extra‐term; LTMSG, reduction sodium long‐term + MSG; XTMSG, reduced sodium extra‐term + MSG.

**TABLE 3:** Ingredient compositions of multigrain bread products

	Ingredient (%) w/w											
Sample	Water	Whole wheat flour	Bread flour	5‐grain cereal	Unsalted butter	Granulated sugar	Nonfat dry milk	Rapid rise yeast	Vital wheat gluten	Vitamin C	Salt	Monosodium glutamate
CTRL	34.32	21.98	20.19	13.46	3.36	2.69	1.93	0.79	0.56	0.08	0.63	0
LT	34.42	22.05	20.25	13.5	3.37	2.7	1.94	0.79	0.56	0.08	0.35	0
XT	34.46	22.07	20.27	13.51	3.38	2.7	1.94	0.79	0.56	0.08	0.24	0
LTMSG	34.41	22.04	20.24	13.49	3.37	2.7	1.94	0.79	0.56	0.08	0.34	0.046
XTMSG	34.44	22.06	20.26	13.51	3.38	2.7	1.94	0.79	0.56	0.08	0.22	0.064
LTMSG2	34.41	22.04	20.24	13.49	3.37	2.7	1.94	0.79	0.56	0.08	0.33	0.046
XTMSG2	34.44	22.06	20.26	13.51	3.38	2.7	1.94	0.79	0.56	0.08	0.22	0.064

Sample abbreviations are as follows: CTRL, full sodium; LT, reduced sodium long‐term; XT, reduced sodium extra‐term; LTMSG, reduced sodium long‐term + MSG; XTMSG, reduced sodium extra‐term + MSG; LTMSG2, reduced sodium long‐term + MSGx2; XTMSG2, reduced sodium extra‐term + MSGx2.

### 2.4 Consumer test protocol

**FIGURE 1:**
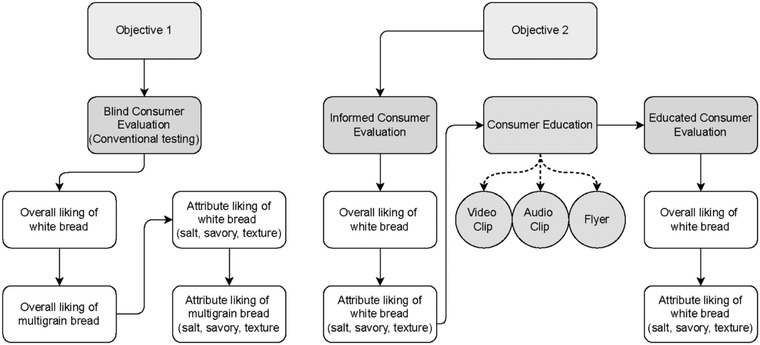
Experimental design of consumer evaluations by research objectives

Consumer testing took place in a sensory lab on the University of Illinois at Urbana‐Champaign campus. Objective 1 was accomplished by conducting consumer evaluations of both multigrain bread and white bread under conventional blind tasting condition. Objective 2 was accomplished by conducting consumer evaluations of white bread under informed condition, followed by consumer evaluations of white bread under education condition. This experimental design is illustrated in Figure 1. Panelists evaluated the samples on their cell phone using an electronic ballot designed using Qualtrics (Provo, UT & Seattle, WA, USA). Ballots were anonymized and treated with confidentiality by study investigators. Panelists received a 1.5″ by 2.5″ portion of each of the coded samples served monadically on 4″ circular white paper plates. A 473‐mL Styrofoam expectorating cup with lid was served along with four unsalted saltine crackers (Kraft Foods Global, Inc., Northfield, IL, USA) in 163‐ml cups and a 473‐ml Styrofoam water cup filled with room temperature water to use for the rinse protocol. Sample order was randomized, and panelists were required to cleanse their palate prior to evaluating their next sample.

In the first session, acceptance was assessed in a conventional consumer test protocol of a blind condition. Panelists first evaluated the five white bread samples for overall acceptance on a 9‐point hedonic scale (Lawless & Heymann, 1999), anchored with 1 = “dislike extremely,” 5 = “neither like nor dislike,” and 9 = “like extremely.” Following overall acceptance of the white bread samples, panelists evaluated the seven multigrain samples for overall acceptance. After overall acceptance of both bread categories, panelists rated each sample on a 9‐point hedonic scale for saltiness liking, savory liking, and texture liking starting with the white bread samples and then the multigrain samples.

In the second session, only white bread samples were evaluated as only one bread variety was needed to investigate the impact of the different educational‐based conditions. The panelists began the evaluations under an informed condition. Panelists were provided information about the sodium content and presence of MSG in each of the samples as part of the sample label to mimic information panelists could obtain from observing a typical nutrition label. Panelists then evaluated their overall liking of the five white breads, followed by their specific attribute liking of the white breads, on a 9‐point hedonic scale with the ends and midpoints anchored.

After concluding the informed evaluations, panelists were given the choice to receive education through either a flyer, an audio clip, or a video clip. Choices in educational format were given to see whether or not consumers had a preference for educational mode and to further see whether consumers with different choices had differing reactions to the samples after education. The educational condition informed panelists of the high incidence of sodium overconsumption, the FDA's voluntary sodium reduction guidelines, and the potential MSG has as a possible solution. Although an average consumer would not receive this level of information by viewing a product and its label, it contains an appropriate level to which could be used in video, audio, or visual advertisements. Each education format took roughly 2 to 3 min to complete and contained the same information as detailed below:

The American Heart Association recommends 1500 mg sodium/day, but 99% of American adults consume levels greater than this recommendation (Cogswell et al., 2012). Extreme sodium intake may lead to hypertension, heart diseases, diabetes, stroke, and kidney diseases (Doyle & Glass, 2010; Havas et al., 2007).

The United States Food and Drug Administration has recently suggested voluntary sodium reductions for packaged, processed, and prepared food. They have set two‐year and ten‐year goals for products including breads and other bakery products. The ten‐year goal reduces sodium content to nearly half of the current sodium value. The reduction in sodium may cause foods to seem less palatable to consumers. Strategies, like the use of flavor enhancers, may address the sodium reduction goals, while still producing desirable products.

The flavor enhancer monosodium glutamate (MSG) has been recognized as safe for consumption for the general population by the United States Food and Drug Administration (Walker & Lupien, 2000; CFR Title 21, n.d.) and has been shown to improve the palatability of reduced sodium products (Roininen et al., 1996). MSG is the sodium salt of glutamic acid, an amino acid, and is naturally present in our bodies. Glutamic acid is naturally present in a variety of foods, including cheese and tomatoes (Center for Food Safety and Applied Nutrition, n.d.). The addition of these flavor enhancers has been shown to make foods more flavorful and savory. In a particular study, sensory panelists were overall more satisfied with the flavor‐enhanced samples and had increased positive emotions when compared to the consumption of a control sample (Miyaki et al., 2015).

The survey had a 3‐min timer embedded to ensure the panelists viewed, listened, or read their selected mode of education prior to proceeding to the educated evaluations. For the informed with education condition evaluations, panelists were provided the same information about the sodium content and presence of MSG as before as part of the sample label and were asked to indicate their liking of samples on a 9‐point hedonic scale with the end‐ and mid‐points anchored first for overall liking, followed by specific attribute liking.

### 2.5 Statistical analysis

Data were analyzed using XL‐STAT 2018.3 (Addinsoft, New York, NY, USA). Analysis of Variance (ANOVA) was used in analyzing the comparison of results among samples and conditions. A three‐way mixed model ANOVA was conducted with condition and sample as fixed factors and panelists as a random factor. The significance level was set at 0.05. Fisher's least significant difference was used for conducting the mean separation test with a 95% confidence interval. Panelists were then grouped according to their overall liking scores based on sample and condition for agglomerative cluster analysis.

## 3 RESULTS AND DISCUSSION

### 3.1 Consumer acceptance for breads with different levels of sodium and flavor enhancers under conventional blind condition

The consumer liking *F*‐values across all conditions for white breads are shown in Table 4. Individual condition results of overall liking are shown in Table 5, and consumer attribute liking results are shown in Table 6. There were significant differences across the samples, and there were differences among panelists as a result of variation in consumer liking. Significant differences in overall liking across the samples were evident. Under conventional blind testing, the full sodium sample (CTRL) was rated highest in overall liking but did not differ significantly from the two samples with a moderate level of sodium reduction (LT and LTMSG). The two samples with the greatest level of sodium reduction (XT and XTMSG) were liked significantly less in comparison to the CTRL and LT samples (Table 5). While other research has found salt content reductions of up to 15% to have no impact on consumer liking of reduced‐sodium white bread, results from this study build upon such findings by indicating that greater levels of sodium reduction can be achieved with no change in consumer liking with either salt removal exclusively or with flavor enhancement (Kuhar et al., 2020). Saltiness liking was no different for the CTRL, LT, and LTMSG samples and were all rated significantly higher for saltiness liking than the XT and XTMSG samples (Table 6). Savory liking followed a similar pattern, in that there were no significant differences in savory liking of the CTRL, LT, and LTMSG samples and they were all rated significantly higher than the XT and XTMSG samples. The CTRL texture was liked most, although not significantly more than the LT sample, and the texture of the XT and XTMSG samples were liked less than the CTRL and LT samples. Although breads with the greatest level of sodium reduction were generally liked significantly less than the full‐sodium bread, a significant reduction in the sodium content of the breads equal to the 2016 FDA's long‐term 10‐year goal of 43% and more than double the reduction goal detailed within the 2021 FDA's updated reduction goals was able to be achieved while being perceived by consumers to be as acceptable as a full‐sodium bread. This could be seen as a marked improvement on prior findings that a 15% to 25% salt reduction should be feasible for most types of bread (Dunteman et al., 2021). Thus, under conventional blind testing, the results of overall liking, saltiness liking, and savory liking suggest that consumers are as accepting of a moderate reduction of sodium in their white bread as they are of a white bread without sodium reduction.

**TABLE 4:** Analysis of variance *F*_calculated_ values for consumer liking across all conditions of white bread

	*F*_calculated_			
Source	Overall liking	Saltiness liking	Savory liking	Texture liking
Condition	0.10	0.45	1.21	1.21
Sample	8.10***	10.30***	11.16***	2.56*
Panelist	3.21***	3.02***	4.43***	7.96***
Condition×Sample	1.34	0.79	2.16*	1.77
Condition×Panelist	1.97***	1.36**	1.46***	1.38**

*, **, and *** indicate significance at *p* < 0.05, *p* < 0.01, and *p* < 0.001, respectively.

**TABLE 5:** Comparison across white bread samples indicated by average overall liking scores within blind, informed, and informed with education conditions

	Mean overall liking^a^				
Condition	CTRL	LT	LTMSG	XT	XTMSG
Blind	6.31 a	6.21 a	5.97 ab	5.76 b	5.31 c
Informed	6.35 a	6.26 a	5.94 ab	5.67 bc	5.36 c
Informed with education	5.86 ab	6.22 a	5.90 ab	5.67 b	5.67 b

*Note*: On a 9‐point hedonic scale, anchored 9 = like extremely, 5 = neither like nor dislike, and 1 = dislike extremely.

Sample abbreviations are as follows: CTRL, full sodium; LT, reduced sodium long‐term; LTMSG, reduced sodium long‐term + MSG; XT, reduced sodium extra‐term; XTMSG, reduced sodium extra‐term + MSG.

^a^ Means showing a common letter across different samples are not significantly different within each condition.

**TABLE 6:** Analysis of variance *F*_calculated_ values for overall liking of multigrain bread in blind condition

	*F*_calculated_			
	Overall liking	Saltiness liking	Savory liking	Texture liking
Sample	2.82*	4.48***	5.48***	1.59
Panelist	4.09***	4.62***	3.64***	6.57***

*, **, and *** indicate significance at *p* < 0.05, *p* < 0.01, and *p* < 0.001, respectively.

The consumer liking *F*‐values for multigrain breads are shown in Table 7. There were significant differences across the samples for overall liking, saltiness liking, and savory liking, and there were differences among panelists as a result of variation in consumer liking. For overall liking, the full sodium sample was rated the highest but did not differ significantly from the two samples with reduced sodium levels without flavor enhancement (Table 8). Apart from XTMSG, reduced‐sodium MSG‐containing samples were overall liked the same as their reduced‐sodium samples without MSG (LT and XT). This suggests that, overall, consumers like a lower level of sodium in their multigrain bread as much as a multigrain bread without sodium reduction. Prior research has found 15% reduced‐sodium multigrain bread without additional reduction strategies to be liked significantly less than the non‐reduced counterpart; in contrast, results from this study suggest incorporating MSG may mitigate palatability loss of reduced‐sodium breads when added salt is limited in the recipe's formulation (Kuhar et al., 2020). Saltiness liking does not differ from the full sodium sample when up to 43% of sodium is reduced regardless of whether MSG is included, indicating saltiness liking is negatively impacted in multigrain breads only when a greater portion of sodium is reduced from the formulation. This is similarly seen in savory liking except for sample LTMSG, where liking of the savory taste is negatively impacted only when sodium reduction is significant. These results suggest that consumers are accepting of multigrain breads with a level of sodium reduction in line with the 2016 FDA's long‐term 10‐year recommended guideline and that inclusion of MSG does not impact salty and savory liking.

**TABLE 7:** Average overall and attribute liking scores of multigrain bread samples (*n* = 78)

	Mean liking score		
Sample	Overall liking^a^	Saltiness liking	Savory liking
CTRL	5.44 a	5.39 a	5.82 a
LT	5.21 ab	5.17 ab	5.47 ab
LTMSG	4.89 bc	5.01 abc	5.03 bc
LTMSG2	4.82 bc	5.27 ab	5.45 ab
XT	5.13 ab	4.83 bcd	4.94 c
XTMSG	4.63 c	4.35 d	4.87 c
XTMSG2	4.74 bc	4.58 cd	4.73 c

*Note*: On a 9‐point hedonic scale, anchored 9 = like extremely, 5 = neither like nor dislike, and 1 = dislike extremely.

Sample abbreviations are as follows: CTRL, full sodium; LT, reduced sodium long‐term; XT, reduced sodium extra‐term; LTMSG, reduced sodium long‐term + MSG; XTMSG, reduced sodium extra‐term + MSG; LTMSG2 reduced sodium long‐term + MSGx2; XTMSG2, reduced sodium extra‐term + MSGx2.

^a^ Means showing a common letter across different samples are not significantly different within each condition.

**TABLE 8:** Analysis of variance *F*_calculated_ values for overall liking across all conditions of white bread for education selection

	*F*_calculated_		
Source	Video clip (*n* = 33)	Audio clip (*n* = 3)	Flyer (*n* = 40)
Condition	0.42	3.15	1.65
Sample	5.80***	2.72	9.07***
Panelist	6.43***	8.26**	11.16***
Condition×Sample	0.68	0.59	1.89
Condition×Panelist	1.56***	1.52	1.74***

*, **, and *** indicate significance at *p* < 0.05, *p* < 0.01, and *p* < 0.001, respectively.

### 3.2 Effect of informed and education conditions

Overall liking ratings within the informed and informed with education conditions are shown in Table 5. Under the informed evaluation condition of the white breads, the full sodium sample was rated the highest but did not differ significantly from the samples with moderate sodium reduction (LT and LTMSG). As there was no condition×sample interaction found, consumers were not significantly influenced by the information. As a result, sample mean ratings remained consistently within 0.1 of a liking score point on the 9‐point scale when comparing between blind and informed conditions (Table 5). While samples in this study had not reached significant differences between the moderately sodium reduced samples and the full sodium sample, the lack of a reduction in liking of samples containing MSG is promising in that consumers may be slowly progressing toward greater acceptance of more well‐known flavor enhancers.

Similar to the informed condition of the white breads, there was no condition×sample interaction found, and thus consumers were not significantly influenced by the information combined with education. As seen in the previous conditions, samples in the informed with education condition were found to have significant differences in liking. The moderately reduced sodium bread sample without flavor enhancement (LT) was found to be liked the most, followed by its MSG‐containing counterpart (LTMSG) and then the full sodium bread, although not significantly. The two samples with the greatest level of sodium reduction (XT and XT+MSG) were both liked significantly less than LT, although not differently compared to LTMSG or the full sodium bread (Table 5). The lack of any statistical difference across breads with greatly reduced sodium and the full sodium bread is promising, as it may indicate that education on the state of sodium consumption can mitigate slight flavor loss from impacting consumer perception. With estimates of fewer than 50% of consumers understanding the relationship between sodium and salt, the nutritional labeling of sodium, and recommended sodium intakes (Ahn et al., 2015), nutritional education may be an effective tool to increase interest in reduced sodium products and has been shown to reduce sodium intake in those receiving the education (Silva‐Santos et al., 2022). In a study in which consumers were provided with bread gradually reducing in salt content, when given in combination with dietary counseling, consumers were found to have increased salt taste sensitivity and greater liking of low salt bread, thus further demonstrating that nutritional education can assist in shifting consumer preferences toward lower sodium foods (Riis et al., 2021). Education positively impacted consumer perception of reduced sodium products containing flavor enhancers where overall liking for certain reduced sodium potato chips and puffed rice snacks increased after education compared to sample liking under blind conditions (Buechler & Lee, 2019). While condition×sample had not reached significance in this study, it could indicate that consumers are placing less importance on clean labeling of their foods than before with neither information nor education differing from their blind evaluations.

### 3.3 Education selection

**FIGURE 2:**
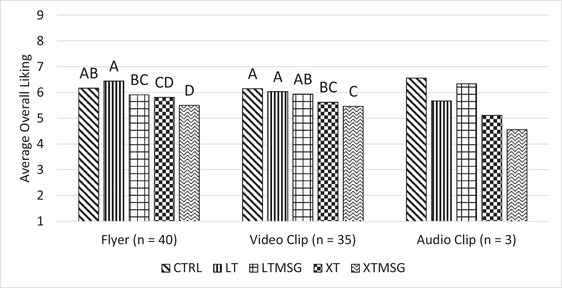
Average liking of white bread samples for three education selections. Means showing a common letter across different samples are not significantly different within each condition. Sample abbreviations are as follows: CTRL, full sodium; LT, reduced sodium long‐term; LTMSG, reduced sodium long‐term + MSG; XT, reduced sodium extra‐term; XTMSG, reduced sodium extra‐term + MSG

Panelists primarily selected either the short video clip (*n* = 35) or the informational flyer (*n* = 40) as their chosen mode of education, with the audio clip rarely selected (*n* = 3). Only samples evaluated under the flyer and video clip selections were found to be significant, although the small number of panelists who selected the audio clip likely limited any differences across the samples to become significant. Consumers who selected the flyer as their choice of education liked the moderately reduced sodium bread most, followed by the full sodium bread, although the difference in liking was not found to be significant. Although the moderately reduced sodium bread with MSG was liked less than its non‐MSG counterpart, it was liked no differently than the full sodium bread. Both breads with significant sodium reduction were liked significantly less than both the full sodium bread and the bread with a moderate level of sodium reduction and no MSG (Figure 2). Consumers who selected the video clip liked the full sodium bread the most, followed by the two moderately reduced sodium breads (LT and LTMSG), although the difference in liking was not significant. Similar to those who selected the flyer, both breads with significant sodium reduction were liked significantly less than the full sodium bread and the moderately sodium reduced bread without MSG (Figure 2).

### 3.4 Consumer segmentation across different conditions

**FIGURE 3:**
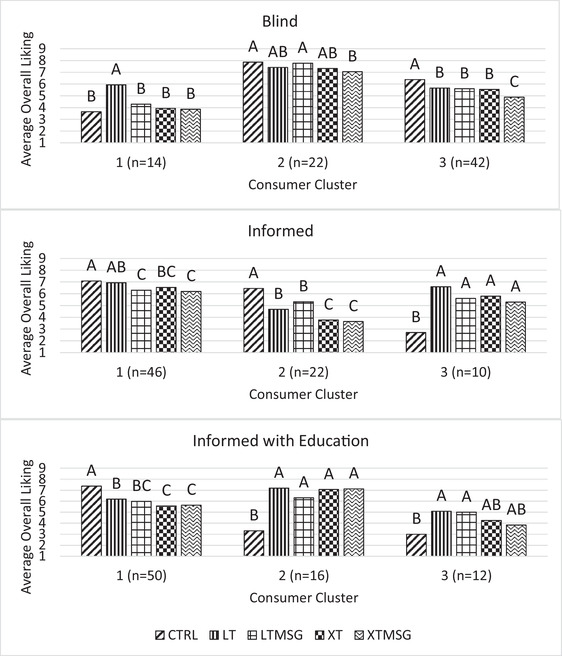
Average liking of white bread samples for three consumer clusters under blind, informed, and informed with education conditions. Means showing a common letter across different samples are not significantly different within each condition. Sample abbreviations are as follows: CTRL, full sodium; LT, reduced sodium long‐term; LTMSG, reduced sodium long‐term + MSG; XT, reduced sodium extra‐term; XTMSG, reduced sodium extra‐term + MSG

Panelists were clustered according to their overall liking ratings across conditions shown in Figure 3. In the blind condition of white bread, panelists who liked a moderate level of sodium reduction (cluster 1, *n* = 14), panelists who rated all samples highly (cluster 2, *n* = 22), and panelists who liked higher sodium (cluster 3, *n* = 42) clusters emerged. Cluster 3 was the largest with over half of the panelists, followed by cluster 2, and last cluster 1.

In the informed condition of white bread, three clusters were identified: panelists who liked all samples but MSG‐containing samples least (cluster 1, *n* = 46), panelists who liked higher sodium (cluster 2, *n* = 22), and panelists who liked reduced sodium breads with or without MSG (cluster 3, *n* = 10). After revealing ingredient information, the number of panelists who liked higher sodium (cluster 3 blind; cluster 2 informed) decreased indicating the influence of sample knowledge of sodium content on reducing the liking of full sodium samples. Additionally, more panelists fell into the cluster 1 (*n* = 46), which liked MSG samples the least, than in the blind condition, possibly indicating that the knowledge of MSG presence may negatively impact sample liking ratings. While analyzing the impact of information on sample liking was not significant when investigating all consumers at once leading to the theory that consumers are progressing away from valuing a clean label, by clustering consumers into groups based on their patterns of liking, it became evident that certain clusters of consumers are still influenced by their perception of clean label ingredients.

In the informed with education condition of white bread, clusters emerged include panelists who liked all samples but preferred higher sodium (cluster 1, *n* = 50), panelists who disliked higher sodium and like reduced sodium breads with or without MSG (cluster 2, *n* = 16), and panelists who like a moderate level of sodium reduction with or without MSG (cluster 3, *n* = 12). The largest cluster consisted of panelists who liked all samples but liking of samples reduced as the sodium content was reduced. From the blind to the informed with education condition, while the number of panelists who preferred higher sodium increased, the number of panelists who liked reduced sodium breads also increased twofold (cluster 1 blind where *n* = 14; clusters 2 and 3 informed with education where *n* = 28).

Food acceptance is heavily impacted by consumer values and ultimately attitudes, which in themselves contain an affective component and an evaluative dimension; these attitudes are not limited to sensory properties and are pertinent to non‐sensory factors incorporated in the foods production processes (Lyman, 1989). It is possible the shift in clusters evident in this study is due to those who previously rated all samples high in the blind condition to feel more comfortable using a larger range of the scale to express their values relating to health consciousness, or a lack thereof, once information about the sample differences was revealed. As white bread is a fairly neutrally flavored product and is often used as a carrier for other foods such as sandwich fillings, consumer's personal consumption habits may have influenced their evaluations; consumers more frequently consuming higher sodium bread toppings or sandwich fillings shifted from liking all samples into a cluster liking sodium‐reduced breads, whereas those who more frequently consumed bread as a standalone food or with naturally low‐sodium toppings or fillings evaluated samples differently knowing the nature of sample differences were related to sodium content rather than another nutritional factor, like caloric or fiber content. While the informed with education clusters may initially appear to provide evidence against MSG inclusion as ratings were not liked significantly more than their non‐MSG‐containing counterparts, the two largest clusters had on average rated reduced sodium breads with and without MSG as greater than five which is the neutral point on a 9‐point hedonic scale, indicating such samples were liked.

**FIGURE 4:**
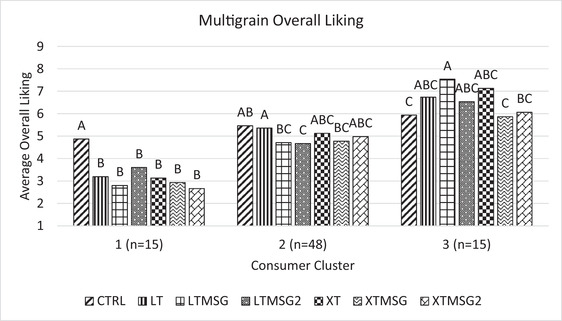
Average liking of multigrain bread samples for three consumer clusters. Means showing a common letter across different samples are not significantly different within each condition. Sample abbreviations are as follows: CTRL, full sodium; LT, reduced sodium long‐term; LTMSG, reduced sodium long‐term + MSG; LTMSG2, reduced sodium long‐term + MSGx2; XT, reduced sodium extra‐term; XTMSG, reduced sodium extra‐term + MSG; XTMSG2, reduced sodium extra‐term + MSGx2

Multigrain bread consumers were clustered according to their overall liking ratings in Figure 4. As multigrain bread was only evaluated under conventional blind condition, this segmentation is based only under such condition. Three clusters emerged which could be described as panelists who disliked all samples but rated higher sodium breads highest (cluster 1, *n* = 15), panelists who rated LTMSG2 only as significantly less liked than the full sodium bread (cluster 2, *n* = 48), and panelists who liked moderately reduced sodium breads with or without MSG and bread greatly reduced in sodium (cluster 3, *n* = 15). Greater than half of the panelists were part of cluster 2, where nearly all reduced sodium breads with or without MSG were liked no different than the full sodium, indicating the largest consumer cluster of multigrain breads were accepting of reductions in sodium content at and greater than those set by the FDA as their long‐term 10‐year goal and more than twice that of the percent reduction goal detailed within the FDA's recently updated sodium reduction guidelines for bread.

## 4 CONCLUSION

Consumer perception of reduced‐sodium flavor‐enhanced bread samples were varied with clusters such as those preferring higher‐sodium samples, those preferring reduced‐sodium breads with MSG, and those who rated all samples similarly. Despite the consumer segmentation, samples with a moderate level of sodium reduction both with and without flavor enhancement by MSG were generally liked similarly to the full sodium bread in regard to overall liking, saltiness liking, and savoriness liking. These results are promising for the feasibility of the FDA's voluntary sodium reduction guidelines, as reduction levels set at their long‐term 10‐year goal were frequently liked the same as a full sodium bread. Furthermore, breads with the largest level of sodium reduction were frequently liked significantly less than all other samples, although it is worth noting that the level of sodium reduction in those samples was more aggressive than the FDA's recommended reductions and could be considered a limitation of this study. These results indicate that breads with a reduced‐sodium content commensurate with the FDA's voluntary guidelines with or without MSG could find success in the market being liked similarly to full‐sodium bread. While consumer education has been previously found to be effective in improving consumer dietary selections and perceptions of flavor additives, no clear impact of information or education on such was evident in our findings. Despite such lack of significance, consumers had a clear preference for receiving education through video clips or flyers over audio clips. This study was limited in the fact that many participants were tied to the university conducting the research, whether by attending as a student or working as staff, and thus the impact of education on the subject may have been minimized had the research included more consumers without higher education. A series of pre‐ and post‐test questionnaires designed to assess participant's knowledge of human nutrition and food additives may have proven useful in determining the extent of this limitation. Further investigation into specific attribute intensities through descriptive analysis would be beneficial in characterizing specific differences across samples and in identifying attributes considered drivers of liking for bread.

## AUTHOR CONTRIBUTIONS

**Dunteman Aubrey**: Conceptualization; Data curation; Formal analysis; Investigation; Methodology; Visualization; Writing – original draft; Writing – review & editing. **Soo‐Yeun Lee**: Conceptualization; Funding acquisition; Project administration; Resources; Supervision; Writing – review & editing.

## CONFLICT OF INTEREST

The authors declare no conflict of interest.
